# Multisensory Integration and Behavioral Plasticity in Sharks from Different Ecological Niches

**DOI:** 10.1371/journal.pone.0093036

**Published:** 2014-04-02

**Authors:** Jayne M. Gardiner, Jelle Atema, Robert E. Hueter, Philip J. Motta

**Affiliations:** 1 University of South Florida, Department of Integrative Biology, Tampa, Florida, United States of America; 2 Mote Marine Laboratory, Center for Shark Research, Sarasota, Florida, United States of America; 3 Boston University, Biology Department, Boston, Massachusetts, United States of America; University of Western Australia, Australia

## Abstract

The underwater sensory world and the sensory systems of aquatic animals have become better understood in recent decades, but typically have been studied one sense at a time. A comprehensive analysis of multisensory interactions during complex behavioral tasks has remained a subject of discussion without experimental evidence. We set out to generate a general model of multisensory information extraction by aquatic animals. For our model we chose to analyze the hierarchical, integrative, and sometimes alternate use of various sensory systems during the feeding sequence in three species of sharks that differ in sensory anatomy and behavioral ecology. By blocking senses in different combinations, we show that when some of their normal sensory cues were unavailable, sharks were often still capable of successfully detecting, tracking and capturing prey by switching to alternate sensory modalities. While there were significant species differences, odor was generally the first signal detected, leading to upstream swimming and wake tracking. Closer to the prey, as more sensory cues became available, the preferred sensory modalities varied among species, with vision, hydrodynamic imaging, electroreception, and touch being important for orienting to, striking at, and capturing the prey. Experimental deprivation of senses showed how sharks exploit the many signals that comprise their sensory world, each sense coming into play as they provide more accurate information during the behavioral sequence of hunting. The results may be applicable to aquatic hunting in general and, with appropriate modification, to other types of animal behavior.

## Introduction

The underwater world provides sensory information that in several ways differs from information signals in an aerial environment: underwater light-scatter severely limits visual distance; the dense aquatic medium allows for about five times faster sound propagation and for subtle hydrodynamic imaging; water propagates electric fields including electromagnetic induction; and odor dispersal remains more coherent as a result of aquatic density stratification [1]. The physical dispersal fields are rather well characterized theoretically, but less is known about the manner in which animals use them (*sensu* Uexküll [2]) in complex tasks and under noisy conditions. Also, sensory perception has been well studied in aquatic animals one sense at a time [3], [4], but the multiple interactions of different senses have remained mostly speculative.

To establish experimentally the multisensory guidance of a complex behavioral task in an aquatic predator, we tested five senses in five phases of hunting behavior in three species of sharks. Because hunting is competitive and strongly correlated with fitness [5], animals are likely to use whichever senses, alone or in combination, that support the best performance [6]. Sensory integration should occur when non-directional signals (odor or sound pressure) can combine with directional signals (hydrodynamic flow). Switching should result when more salient signals appear (e.g. closer to the signal source) as the animal moves from one behavioral phase to the next [7]. If alternate senses can provide information useful to the behavioral task, these may be used when environmental conditions change (e.g. nighttime and turbidity reduce visual resolution [8]), when a sense organ becomes damaged (e.g. by disease or chemical pollutants [9]), or when sensory cues become masked (e.g. by boat noise [10]).

Hunting involves: 1) initially *detecting* and evaluating cue(s) that alert the hunter to the presence of prey somewhere, 2) *tracking* the cues to the vicinity of their source, 3) *orienting* to the prey with direct sensory contact, 4) *striking* at the prey, and 5) coordinating strike behavior with jaw and/or appendage motion to *capture* the prey [11]. The timing of these hunting phases accelerates from minutes (tracking) to milliseconds (capture) and various senses guide them. While a single sensory modality may suffice for some behaviors, information from multiple cues can result in shorter latency, greater sensitivity, better spatial and temporal resolution, and improved noise rejection [12].

Sharks capture prey in a variety of ways, such as ram, suction, and biting. In pure suction feeding, the predator remains completely stationary as it rapidly expands the buccopharyngeal cavity to draw the prey into its mouth. In pure ram feeding, the predator accelerates to overtake and engulf a completely stationary prey. Most fishes fall somewhere in the middle of the spectrum. In ram-biting, rather than completely engulfing the prey, the shark will bite into the prey (reviewed in [13]). All capture modalities require sensory control to precisely time and direct jaw movements.

**Figure 1:**
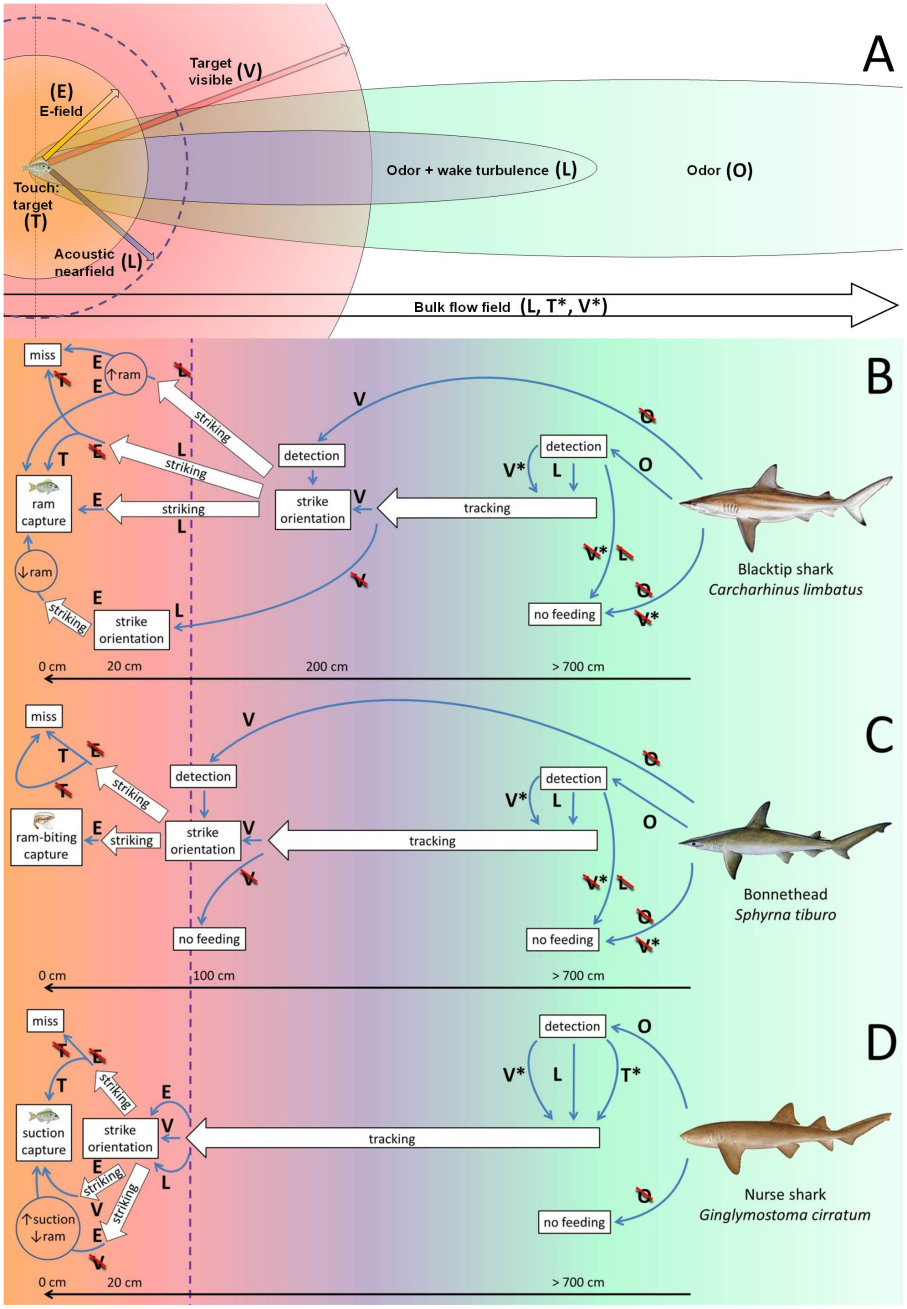
Sensory signals and their use by hunting sharks. Senses are indicated by capital letters (e.g. V = vision); asterisk (e.g. V*) denotes sense used to measure and orient to the bulk flow (i.e., by detecting environment features); no asterisk (e.g. V) denotes sense used to process prey cues; slashed (e.g. 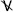) indicates sensory block. Background colors in A, B, and C indicate areas of signal availability corresponding to signal dispersal fields in A. Behavioral phases in boxes occur at a discrete distance from the source; behavioral phases in boxed arrows occur over a distance; line arrows indicate transitions from one phase of the behavior to the next. **A. Physical model of prey signal fields** (After [1]). Prey emit a complex mixture of sensory stimuli that radiate and disperse into the habitat. Animals can detect the bulk flow vector (arrow) by measuring their drift along the walls and substrate, using vision (V*) or touch (T*) of the walls or bottom, or, by detecting turbulence in the bulk flow, with the lateral line (L). Bulk flow disperses prey odor downstream over large distances where it can be detected by olfaction (O, green); closer to the source, prey-generated wake turbulence becomes detectable by the lateral line (L, purple). Close to the source, the prey becomes directly detectable based on vision (V, red), lateral line imaging of the acoustic near field (L, delineated by purple dotted line), electroreception (E, orange), and touch (T, direct tactile contact with prey). **B. The blacktip shark,** ***Carcharhinus limbatus*** **.** From downstream, the blacktip shark detects the presence of prey using O and, during the daytime, tracks the bulk flow upstream using OV* or OL. Seeing the prey, it switches to V to orient and strike from a distance (∼2 m). Near the prey, the strike is adjusted using L. Then it switches to E to ram-capture the prey. With the lateral line blocked (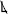) it often misses the prey; successful captures involve increased ram. If 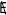, it can capture prey using T; if 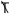, it will miss. When approaching prey from downstream at night (under moonless conditions; 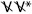), it detects (O) and tracks (OL) the prey until it is at close range (∼20 cm), then orients and strikes using L, but captures using less ram. If 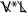, it detects the prey (O), but cannot track and ceases to feed. When approaching prey from upstream (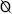), it detects the prey using V and orients, strikes, and captures. If it approaches the prey from upstream at night (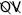), it will not detect the prey and will not feed. **C. The bonnethead,** ***Sphyrna tiburo*** **.** From downstream, the bonnethead detects prey using O and, during the daytime, tracks it using OV* or OL; it switches to V to orient and strike, but does so at a closer range (∼1 m) than the blacktip shark, then switches to E to capture using ram-biting. When approaching prey from downstream at night (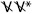), it detects (O) and tracks the prey (OL), but cannot orient or strike and ceases to feed. If 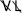, it detects prey (O), but cannot track, and ceases to feed. When approaching prey from upstream (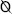), it detects prey using V, then orients, strikes, and captures. At night (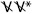), it detects (O) and tracks (OL) prey, but cannot orient and strike and ceases to feed. If 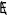, it misses the prey even when touching it (T or 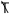). **D. The nurse shark,** ***Ginglymostoma cirratum*** **.** From downstream, the nurse shark detects prey using O, then, during the daytime, tracks using OV*, OL, or OT*. At a close range it switches to V, L, or E to orient and strike, then switches to E to suction-capture the prey. At night (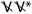), it detects (O), tracks (OL or OT*), orients and strikes (L or E) as above, but modulates its capture by increasing suction and decreasing ram. When approaching prey from upstream (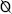), it does not detect the prey and does not feed. Like the blacktip shark, if 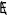, it can still capture the prey if it touches it (T), but it misses when it does not touch (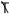) the prey. Nurse shark illustration copyright José Castro, with permission. Pinfish, shrimp, bonnethead, and blacktip shark illustrations copyright Diane Peebles, with permission.

We chose, in a size range (∼1 m total length) conducive to controlled laboratory studies, three species of sharks from different ecological niches and with different capture modalities (Figure 1): ram-feeding blacktip sharks, *Carcharhinus limbatus*, that rapidly chase down midwater teleost prey [14]; ram-biting bonnetheads, *Sphyrna tiburo*, that scoop crustaceans off seagrass beds [15]; and benthic, suction-feeding nurse sharks, *Ginglymostoma cirratum*, that hunt nocturnally for crustaceans and fish, often sucking them out of reef crevices [16]. By blocking various senses, singly and in combination, we learned their involvement in guiding the different hunting phases.

**Figure 2:**
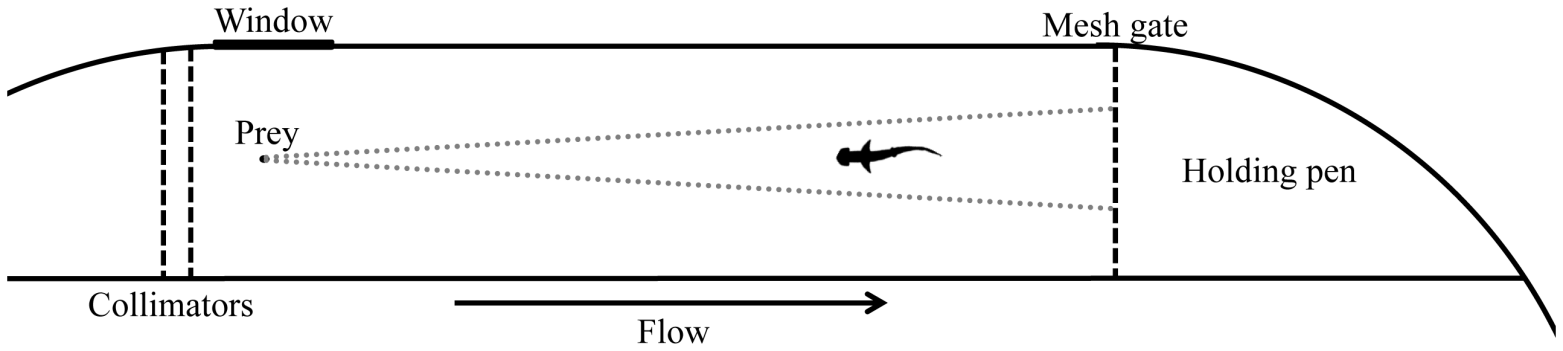
Experimental flume setup. Top view diagram, to scale, with a ∼1 m bonnethead in the test arena, swimming up a plume (approximate outline indicated by gray dotted lines) emanating from the prey. The black dot represents the location of the prey only and is not representative of size. The window allows a side view of the prey area for observation and high speed video recording of the strike-capture sequence. The upstream collimators create uniform, small-scale turbulence and uniform flow through the flume. The downstream gate can be raised to release the shark from the pen used to hold each animal prior to testing. The large remaining part of the oval tank (shown partially) was used to maintain experimental animals.

As a realistic multimodal signal source we used a small (∼8 cm), live prey tethered upstream at 60 cm (blacktip shark), 30 cm (bonnethead), or 10 cm (nurse shark) above the bottom in the center of the (1.2 m deep) water column of a 2 m×7.5 m flume channel. Generalized, prey-generated, aquatic signal fields are diagrammed in Figure 1A. Under natural conditions, bio-electric fields are detectable at a distance of less than one-half meter from the source [17]. The maximum range of detection of hydrodynamic images (the acoustic near-field detected by the lateral line and vestibular organs) is 0.4–2 predator body lengths from the source [18]. Visual detection distance rarely exceeds tens of meters [8]. While far-field sound pressure signals, particularly low frequency signals, may be detectable over distances of (many) kilometers, source *direction* may be detectable over only tens to hundreds of meters [19]. Wakes with source-generated odor and turbulence signals can be carried by bulk flow over great distances from the source. Beyond that remains a rather non-directional odor far-field that signals the presence of a (food) source. Indeed, odor is often the first cue encountered by aquatic animals searching for food (reviewed in [1]). We characterized the prey odor plume in independent dye tests with similar flow conditions (Figure 2). Tank studies preclude the useful analysis of directional sound. Signal detection distances are dependent on source strength, wavelength and environmental signal-to-noise ratios; these conditions were standardized across all tests.

The goals of this study were to: 1) examine the integration of information from the olfactory, mechanoreceptive, visual, and electroreceptive senses at each stage of the feeding sequence in sharks; 2) investigate sensory switching; and to 3) elucidate the complementary and alternative roles of the senses in each phase of feeding behavior. Examining three shark species from different habitats, with different feeding strategies for different prey types, allowed us to compare, under similar testing conditions, the use of various senses in animals adapted to the ecology of disparate environments.

## Materials and Methods

### Ethics statement

Shark collections were conducted with permission from the Florida Fish and Wildlife Conservation Commission (07SR-041B and SAL-10-0041-SRP). This study was carried out in accordance with protocols approved by the Institutional Animal Care and Use Committees at the University of South Florida (W3817) and Mote Marine Laboratory (11-03-RH1).

### Experimental animals

Eighteen young-of-the-year (YOY) blacktip sharks, *Carcharhinus limbatus*, 51–65 cm total length (TL), were collected from Terra Ceia Bay on the southwest Florida coast using rod and reel and gillnet gear. Ten juvenile nurse sharks, *Ginglymostoma cirratum*, 67–94 cm TL, were collected from waters near Long Key in the Florida Keys using rod and reel gear. Sixteen bonnetheads, *Sphyrna tiburo*, 69–95 cm TL, were collected from Terra Ceia Bay and waters near Sarasota, Florida using gill net gear. Sharks were transported to Mote Marine Laboratory in Sarasota where they were held in a 210,000 L oval tank operated on a closed recirculating life support system with sand filtration and heater/chiller units, maintained 24–26°C on a 12 h:12 h light:dark cycle. Animals were fed fish, shrimp, and squid, supplemented with Mazuri Vita-Zu Sharks/Rays Vitamin Supplement Tablets (PMI Nutrition International, St Louis, MO, USA), to satiation three times per week, except during periods of experimentation, when food was withheld for 48 hours prior to behavioral trials to ensure that the animals were motivated to feed.

### Behavioral procedures

Experiments were conducted in a near-laminar flow channel (flume) constructed within the 210,000 liter oval tank; working area (test arena) was 7.5 m long×2 m wide, filled to 120 cm depth, with a flow rate of 2.3 cm/s (Figure 2). Dye tests showed uniform flow with boundary layer shear near the walls and bottom. A 4 m×2 m holding area at the downstream end served as an animal containment area behind a mesh gate. As per Gardiner and Atema [20], for each trial, an individual animal was moved into the flume channel and allowed to acclimate for 30 minutes, then offered a small piece of food to confirm that it was hungry. The animal was then herded into the holding pen. A live prey item from the diet of each species (nurse and blacktip sharks: pinfish, *Lagodon rhomboides* [21], [22]; bonnethead: pink shrimp, *Farfantepenaeus duorarum* [23]), was tethered at the upstream end of the flume using a piece of thin, degradable cotton thread, inserted through the musculature. The prey were, therefore, injured from the tether attachment. This tether restricted the prey to the area in front of a window in the side of the tank. Since prey size can affect capture kinematics [24], the prey items were size-matched to the total length of the shark and prey size was consistent across trials. Prey was suspended midwater, at 60 cm (blacktip sharks), 30 cm (bonnetheads) or 10 cm (nurse sharks) above the bottom. Independent dye studies showed the shape and extent of the odor/turbulence plume emanating from the prey (Figure 2). The shark was held in the start box for six minutes to allow a plume of sensory cues emanating from the prey to establish along the length of the flume channel. The shark was then released and a trial proceeded for 10 minutes or until the prey was consumed, during which time the shark's behavior was simultaneously monitored and filmed from above using a series of three overhead cameras (Sony 1/3 inch CCD Camera, Model CUC8752, CIB Security, Sunnyvale, CA, USA). A lateral view of any strikes or bites on the prey was recorded using a fourth camera placed in front of the previously mentioned window in the tank wall (Figure 2). The images from these cameras were combined using a multiplexer (Nuvico EV-8250N, Englewood, NJ, USA) and saved digitally via a computer. Additionally, any bites or strikes were filmed laterally at 250 frames/s using a Photron FASTCAM-X 1024 PCI Model 100 K camera (Photron USA Inc., San Diego, CA, USA), which was also placed in front of the window in the tank wall (Figure 2). Animals were examined intact and after blocking each of the sensory systems (outlined below), alone and in combination.

### Sensory deprivation

Olfaction was blocked by inserting pieces of cotton soaked in petroleum jelly into the animal's nares [25]. To block vision, the eyes were covered with small pieces of heavy black plastic, attached to the skin around the margins of the eyes with cyanoacrylate glue. The sensitivity of the electrosensory system was reduced by painting over the pores of the ampullae of Lorenzini with cyanoacrylate glue (blacktip and nurse sharks; The Original Super Glue, Super Glue Corp., Rancho Cucamonga, CA USA) or silicone-rubber paint (bonnetheads; Smooth-On Mold Max Stroke, Smooth-On Inc., Easton, PA USA). The location of the pores in these species has previously been mapped [26], [27]. Prior to use on animals, the insulating nature of these two materials was verified by covering one electrode on the prey-simulating electrical stimulus apparatus described by Kajiura and Holland [17]. The pair of electrodes was then immersed in seawater and a current of up to 200 mA was applied; no current was detected at the multimeter, indicating that the paint and glue break the electrical circuit and are therefore insulating. To minimize any distress, all of these blocks were applied while the animal was under anesthesia with tricaine methanesulfonate (MS-222), with a dose of 100 mg/L in buffered seawater for induction and 50 mg/L for maintenance. Animals were ventilated using a hose attached to a small recirculating pump while the blocks were applied, then revived using fresh seawater. Animals were allowed to recover in approximately 1000 L of seawater in a 244 cm diameter round tank for three hours, then moved to the flume channel and allowed to acclimatize for a further 30 minutes as above, prior to a behavioral trial. MS-222 is a sensory depressant requiring 1.5 hours of recovery [28].

The lateral line system was lesioned by holding the animals in a 0.5 g/L solution of aerated streptomycin sulfate in seawater for three hours [20]. An individual animal was held in approximately 1000 L of this solution in the 244 cm diameter round tank. For combinations of sensory blocks, those requiring anesthesia were first applied, then the animal was moved to the recovery round tank where it was held until it had recovered sufficiently to swim and navigate the tank normally. The streptomycin sulfate was added to the water and the animal was held in this solution for three hours as described above, prior to being moved to the flume channel for behavioral testing. Streptomycin is an ototoxic antibiotic that has been shown to lesion both the surface neuromasts and canal neuromasts in teleosts [29]. In amphibians, treatment with this drug results in an increase in spontaneous firing of the afferent nerves, which is linked to direct effects on the membrane of the hair cell, and a large lag phase in the receptor potentials, which may be caused by interference with the motion of the sensory hairs [30]. It does not affect inner ear function unless applied intralumenally [31]–[33]. The duration of the effects of this drug is not completely understood. Since teleosts treated with this drug return to normal behavior in 20–24 hours [34], all lateral line blocked trials were completed within six hours of application of the drug. However, since physical damage to the hair cells has been found on scanning electron micrographs of streptomycin-treated neuromasts [32], [33], following lateral line lesion treatments, animals were allowed to recover for a minimum of four weeks prior to any other behavioral testing to allow time for the neuromasts to regenerate [35], [36]. While hearing with the inner ear may be contributing to prey localization in sharks [37], [38], the stimulus field in a closed tank environment is very difficult to control due to ambient noise, as well as echoing off the walls, bottom and surface of the tank, and thus it was not specifically examined in this study.

### Video analysis

All videos were digitized using MaxTRAQ Lite+ v.2.2.2.2 software (Innovision Systems Inc., Columbiaville, MI, USA). As described above, the behavior of these animals can be divided into five phases: detection, tracking, orientation, striking, and capture. **Detection** is indicated by the initiation of feeding behavior (i.e. the onset of tracking, or in the absence of tracking, the onset of orientation/striking). **Tracking** in other shark species typically begins with a rapid turn and descent towards the bottom, followed by tight circles and figure-8 patterns as the animal approaches the prey from downstream [20], [39]–[43]. **Orientation**, a turn to align the body or head for the strike, is immediately followed by striking. **Striking** in ram-feeding bony fish is defined as direct, rapid whole-body acceleration towards the prey, often using an S-start [44]. In fishes, **capture** begins with the onset of jaw depression [13], [45] (i.e. mouth opening) and for comparative purposes, in this study was defined as ending when the center of mass of the prey had passed the anterior margin of the mouth.

In intact animals, detection is typically indicated by the start of tracking behavior, which ends with orientation (a turn) and a directed strike that culminates in capture. From the composite view of the three overhead cameras and lateral camera, the onset time of each of these behaviors was noted. For the tracking phase, the following variables were examined: 1) swim velocity, in body lengths/s; 2) turn velocity, in °/s; 3) frequency of turns, in turns/s; and 4) tracking time, in s, from the start of tracking to the first strike. To account for differences in the distance at which tracking began, tracking time was standardized by dividing it by predator-prey distance at the onset of tracking, expressed as a proportion of the total length of the test arena, i.e., time_std._  =  time/(distance to prey/test arena length). For the orientation, striking, and capture phases, 5) orientation distance, predator-prey distance at orientation, in cm; 6) strike rate, percentage of trials in which strikes occurred; 7) strike angle, in °, the angle between the midline of the predator and center of mass of the prey; 8) strike velocity, in body lengths/s; 9) number of misses; and 10) capture success rate, percentage of trials resulting in successful capture, were examined.

### Statistical Analyses

Data for each species were regressed against total length using the least squares method to remove the effects of size [46] and the standardized residuals were used in all subsequent analyses. All data were tested for normality and equality of variance with Kolmogorov-Smirnov tests and Levene Median tests, respectively [47]. Florida Fish and Wildlife Conservation Commission regulations prohibit the release of any fishes that are held in captivity for more than 30 days or treated with any chemicals and so, in an effort to reduce the number of animals taken from the wild, individual animals were used in more than one, but not all, treatment groups resulting in an unbalanced design. Data for the different treatments were therefore compared within each species using linear mixed models. Non-parametric repeated measures analyses were conducted using Skillings-Mack tests. When significant differences were found, Tukey post hoc tests were then used to perform pairwise comparisons of the treatments. The Benjamini-Hochberg method was used to control the false discovery rate in multiple statistical tests [48]. Analyses were conducted using R (version 3.0.2, [49]), *nlme* (version 3.1-111, [50]), *multcomp* (version 1.3-1, [51]), *Skillings-Mack* (version 1.0-2, [52]), and *nparcomp* (version 2.0, [53]).

## Results and Discussion

To guide the reader through the analysis of results from multiple sensory blocks affecting five phases of hunting in three species (Figure 1), we start with a generalized description. Based on current and previous results, the hunting sequence can be described as follows. The shark cruises out of the gate; it changes swimming behavior (more frequent and faster turns, but no change in swimming velocity) indicating odor detection and the start of tracking. While odor *per se* is non-directional and concentration gradients in odor plumes are too chaotic to provide useful directional information, odor is eminently suited for prey identification and motivating subsequent behavior [1], [54]. At the farthest detection distance the sharks have only patches of odor available (odor far-field), followed by the addition of wake turbulence (odor near-field). Then, using both odor and lateral line information [20], the shark starts tracking the plume. To stay connected with the plume they steer into concentration patches based on detection of sub-second time differences between bilateral odor encounters [55]. Upon visual contact with the prey, ram-feeding sharks orient and accelerate into a strike, where the prey's hydrodynamic field guides the precise directional and temporal coordination of swimming and mouth positioning. Suction-feeding sharks track until in close proximity, then visual, electrical, hydrodynamic or tactile cues prompt them to strike by raising the head. In all species, electric fields guide the timing of jaw opening with millisecond precision. Experimental evidence below follows the hunting sequence from detection to capture. Data for all treatments are presented in Tables S1–S3.

### Detection

Despite their different sensory specializations, when approaching prey from downstream, all three shark species detected the distant presence of prey by olfaction. With olfaction blocks, blacktip sharks and bonnetheads kept cruising and did not track the plume, but at much closer range (2 m and 1 m respectively) detected prey visually and proceeded to strike and capture. With olfaction *and* vision blocked simultaneously, neither blacktip sharks nor bonnetheads detected the presence of prey and thus failed to feed (Figures 1B–D, green area). Nurse sharks absolutely required odor: blocking olfaction abolished detection and feeding. Olfactory-blocked nurse sharks spent a significantly greater proportion of their time resting on bottom [intact: 0.2±0.2%, olfaction blocked: 50.9±12.2%, *P*<0.001].

Although the nurse shark possesses retinal areas specialized for enhanced visual acuity [56], [57], and vision is clearly important for other behaviors [58], they do not appear to identify prey visually. This species has been described as a nocturnal hunter, often cornering fish in reef crevices at night [16], [59], [60]. Visual cues may be diminished on a dark night or even unavailable in the case of hidden prey, which may explain why chemical cues are more important than visual cues for feeding in this species. The smooth dogfish, *Mustelus canis*, a crepuscular hunter, also requires olfaction to detect prey [25]. Many shark species approach their prey from downstream [20], [39], [40] and olfaction has long been thought to be the primary sensory modality for prey detection [61]. However, our results demonstrate that at least some species can detect prey visually, suggesting that they could also approach prey from upstream (odor cues are unavailable as they are carried away from the prey by the flow), provided there is good visibility (i.e., daytime hunting with good to moderate water visibility). While bonnetheads are thought to be diurnal hunters [62] and blacktip sharks primarily crepuscular [63], their inability to detect prey in the absence of odor and vision suggests that if they hunt at night, they likely approach prey from downstream.

### Tracking: rheotaxis in bulk flow and eddy chemotaxis in wakes

After olfactory-based detection, navigation to the vicinity of an odor source is based on upstream swimming and wake tracking [20], [59], [60]. Orientation to flow is often referred to as “rheotaxis” [64]. The bulk flow vector (Figure 1A) needed to steer upstream swimming is *not* source-directed and animals can determine it only by measuring their drift against an external frame of reference, typically by seeing or touching fixed structures such as the walls or bottom [64], or by detecting turbulence contained in the bulk flow [65], such as the shear/turbulence found at boundary layers near the walls and bottom. Famously, moths in pheromone plumes steer by the visual flow field [66]. Tracking the odor-flavored eddies of a source-generated wake has been called “eddy-chemotaxis” [1] to distinguish it from rheotaxis.

**Figure 3:**
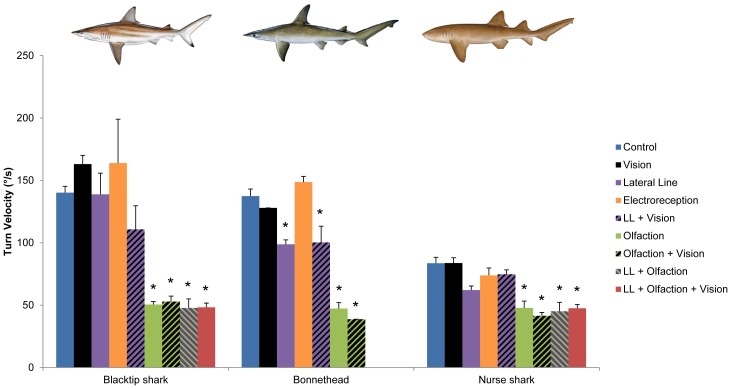
Turn velocity during tracking. The turning velocity, during the tracking phase, in three species of sharks, the blacktip shark, *Carcharhinus limbatus*, the bonnethead, *Sphyrna tiburo*, and the nurse shark, *Ginglymostoma cirratum*, with all senses intact (control) and following blocks of the sensory systems indicated in the figure legend (LL: lateral line). Error bars are ± s.e.m. * denotes treatments that are significantly different from control at α = 0.05; for comparisons among treatments, see Tables S1–S3. Nurse shark illustration copyright José Castro, with permission. Bonnethead, and blacktip shark illustrations copyright Diane Peebles, with permission.

**Figure 4:**
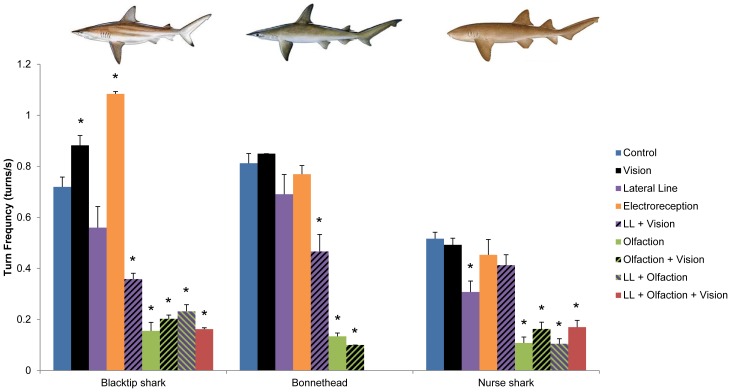
Turn frequency during tracking. The frequency of turns (turns/s), during the tracking phase, in three species of sharks, the blacktip shark, *Carcharhinus limbatus*, the bonnethead, *Sphyrna tiburo*, and the nurse shark, *Ginglymostoma cirratum*, with all senses intact (control) and following blocks of the sensory systems indicated in the figure legend (LL: lateral line). Error bars are ± s.e.m. * denotes treatments that are significantly different from control at α = 0.05; for comparisons among treatments, see Tables S1–S3. Nurse shark illustration copyright José Castro, with permission. Bonnethead, and blacktip shark illustrations copyright Diane Peebles, with permission.

Tracking behavior in other shark species has been described as tight circles and figure-8 patterns [20], [39], [40], [43]. In our study, we have defined it as a period of high-velocity, high-frequency turns (blacktip shark, control: 140.2±2.1°/s, 0.72±0.04 turns/s; bonnethead, control: 137.4±5.7°/s, 0.81±0.04 turns/s; nurse shark, control: 83.6±4.8°/s, 0.52±0.03 turns/s; Figures 3 and 4). Swimming velocity varied slightly among the treatments. Blacktip sharks swam slower when olfaction and vision were blocked (olfaction + vision blocked: 0.72±0.03 BL/s, *P*<0.001; lateral line + olfaction + vision blocked: 0.73±0.08 BL/s, *P* = 0.009; lateral line + vision: 0.70±0.04 BL/s, *P*<0.001). Bonnetheads swam slower with lateral line and vision blocked (0.49±0.03 BL/s, *P* = 0.009). Nurse sharks swam faster with lateral line blocked, alone or in combination with olfaction (lateral line blocked: 0.76±0.07 BL/s, *P*<0.001; lateral line + olfaction blocked: 0.73±0.08 BL/s, *P* = 0.009). In the smooth dogfish, *M. canis*, tracking requires simultaneous olfactory and hydrodynamic cues [20]. This species can orient to the bulk flow and navigate upstream using either vision or the lateral line, but it requires lateral line input to follow the odor-flavored wake. Sensory blocks in the blacktip shark and bonnethead showed their tracking and upstream swimming were also dependent on olfaction in combination with either vision or the lateral line. In all three species, when olfaction was blocked, alone or in combination with other senses, turns were significantly slower and less frequent compared to the unblocked condition (blacktip shark, olfaction blocked: 54.3±2.3 °/s, *P*<0.001; 0.16±0.03 turns/s, *P*<0.001; bonnethead, olfaction blocked: 47.2±4.9 °/s, *P*<0.001; 0.13±0.01 turns/s, *P*<0.001; nurse shark, olfaction blocked: 47.8±2.9 °/s, *P*<0.001; 0.11±0.01 turns/s, *P*<0.001; Figures 3 and 4); this behavior is similar to that of a shark that simply cruises the tank in the absence of prey and suggests that odor motivates the behavior. When vision and the lateral line were simultaneously blocked, blacktip sharks and bonnetheads turned quickly, but infrequently (blacktip shark: 110.8±18.9 °/s, *P*>0.05; 0.36±0.03 turns/s, *P*<0.001; bonnethead: 100.3±13.0 °/s, *P* = 0.03; 0.47±0.07 turns/s, *P*<0.001; Figures 3 and 4), and could not locate the prey, indicating that vision or the lateral line provide the directional vector required for source localization, as in *M. canis* [20]. The nurse shark, on the other hand, could continue to track and successfully locate prey when both vision and the lateral line were blocked (74.8±3.6 °/s, *P* = 0.984; 0.41±0.04 turns/s, *P* = 0.900; Figures 3 and 4). Since this species tends to maintain contact with the bottom as it swims, occasionally even using its pectoral fins to propel itself [67], it appears to use tactile substrate cues with free nerve endings in the skin [68] to measure drift and to orient to the flow. Tactile orientation to flow has been described in teleost fish [64], [69], [70] and suggested to be possible for the epaulette shark, *Hemiscyllium ocellatum* [71], but this is the first evidence of the use of tactile cues for tracking. However, reaching the prey with olfaction and touch is a slow and convoluted process (intact: 98.4±20.6 s, vision and lateral line blocked: 189.8±79.1 s, *P*<0.001).

### Strike/orientation

**Figure 5:**
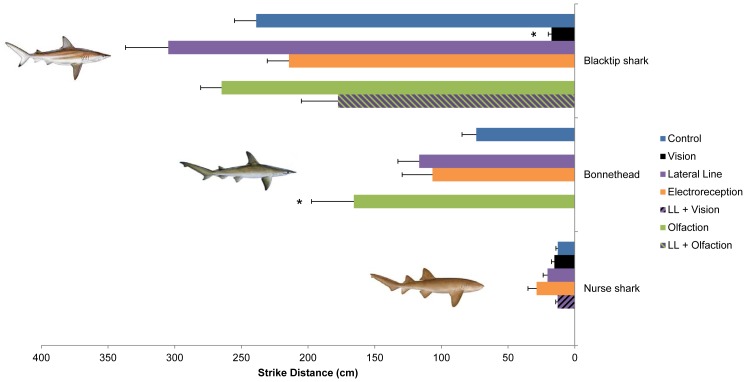
Strike distance. The distance between the predator and the prey at the initiation of the strike, in cm, in three species of sharks, the blacktip shark, *Carcharhinus limbatus*, the bonnethead, *Sphyrna tiburo*, and the nurse shark, *Ginglymostoma cirratum*, in animals with all senses intact (control) and following blocks of the sensory systems as indicated (LL: lateral line). Treatments in which striking did not occur have been omitted. Error bars are ± s.e.m. * denotes treatments that are significantly different from control at α = 0.05; for comparisons among treatments, see Tables S1–S3. Nurse shark illustration copyright José Castro, with permission. Bonnethead, and blacktip shark illustrations copyright Diane Peebles, with permission.

Once an animal had tracked the odor plume to the vicinity of the source, or, in the absence of tracking, upon visual detection (blacktip shark or bonnethead), striking immediately followed. Strikes are fast and need to be precisely oriented. Sensory control of orientation and striking behaviors differed among the three species. Intact blacktip sharks oriented from a distance of a few meters (238.9±16.3 cm; Figure 5) and executed direct (angle of 15.4±2.5° to the prey), rapid strikes (111.0±11.9 cm/s, equivalent to 1.95±0.21 Body Lengths per second, BL/s). Significantly fewer vision-blocked animals struck (60.4±11.1%, *P*<0.05) and only after lengthy search times (intact: 35.0±9.5 s, vision blocked: 949.3±236.1 s; *P*<0.001). Vision blocked strikes occurred only from a distance of a few centimeters (17.4±2.5 cm, *P*<0.001; Figure 5), from greater angles (91.7±15.5°, *P*<0.001), and at reduced velocity (70.3±17.4 cm/s or 1.25±0.31 BL/s, *P* = 0.001), indicating that orientation of the long distance strikes is visually guided. In the absence of vision, strikes were guided by the lateral line, as animals with simultaneous vision and lateral line blocks did not orient or strike, even when they were within electrosensory prey detection range [17]. This also suggests that electrical cues alone were not sufficient to prompt a strike. In other studies of the role of electroreception in predation, olfactory stimuli have been required for the initiation of feeding behavior [17], [43], [72]–[80]. Collectively, these results suggest that sharks do not recognize electrical cues alone as prey, but require an additional visual or olfactory cue.

Intact bonnetheads, compared to blacktip sharks, oriented in closer proximity (73.8±10.7 cm; Figure 5) and a greater angle (28.1±5.1°) to the prey, and struck with lower velocity (59.2±2.34 cm/s, equivalent to 0.70±0.03 BL/s). Bonnethead orientation and striking appeared entirely visually guided: blinded animals never successfully executed a strike, even when lateral line cues, which could guide striking in blacktip sharks, were available. Olfaction-blocked animals began the visually guided strike from a greater distance to the prey, compared to the control treatment (165.7±31.8 cm, *P*<0.001; Figure 5) but at a similar angle (26.5±8.9°) and with a similar velocity (0.84±0.04 BL/s). Striking behavior was not significantly affected by any other treatment. Both blacktip sharks and bonnetheads are ram-feeders [14], [15]. Ram feeding involves the predator overtaking its prey with the mouth open, which inherently requires that the predator pinpoints its prey from a distance in order to have sufficient room to accelerate [44]. Vision provides the best performance for this task. It allows the animal to precisely localize the prey from a greater distance than the lateral line, which functions over distances of 0.4–2 predator body lengths [18], [81]. Electroreception alone cannot mediate orientation and striking in these species, perhaps because it functions only over distances of tens of centimeters [17], [82]. Hammerhead sharks, such as the bonnethead, possess enhanced binocular vision compared to pointed-nose sharks, such as the blacktip shark [83], which may explain the bonnethead's reliance on vision for striking at prey in the water column.

Intact nurse sharks orient from a close proximity than blacktip sharks or bonnetheads (12.7±1.4 cm; Figure 5), and greater angles (58.4±10.4°), striking with a slower velocity (26.1±3.39 cm/s, equivalent to 0.31±0.04 BL/s). Vision, lateral line, or electroreception blocks did not cause significant changes in either the frequency of orientation and striking [all treatments: 100%], or in striking distance [vision blocked: 15.2±2.2 cm, lateral line blocked: 20.4±3.3 cm, electroreception blocked: 28.6±6.5 cm, *P* = 0.328; Figure 5]. Strike angle, however, was significantly greater after lateral line + vision blocks or electroreception blocks [lateral line + vision blocked: 91.9±22.8°, electroreception blocked: 93.7±6.3, *P* = 0.006]. Strike velocity was slower when vision was blocked and faster when the lateral line or electroreception was blocked [vision blocked: 0.23±0.06 BL/s, vision + lateral line blocked: 0.23±0.03 BL/s, lateral line blocked: 0.44±0.06 BL/s, electroreception blocked: 0.44±0.04 BL/s, *P* = 0.001]. Since suction feeding is only effective over short (cm) distances [16], [84], suction feeders (e.g. nurse sharks) can use any of these senses to successfully align their short-distance strikes, although there are slight differences in the strike, depending on the sensory modality used. The Pacific angel shark, *Squatina californica*, a lie-in-wait ambush predator that is believed to be a suction feeder, can also use vision or mechanoreception to align short distance (10 cm) strikes; electroreception has not yet been examined [85].

Ram-feeding teleosts typically brake (decelerate) just before capture, which has been suggested to increase capture accuracy by allowing more time for steering and positioning [86], [87]. The lateral line mediates this behavior in largemouth bass, *Micropterus salmoides* [44], either by providing the animals with information on the position of the prey, just prior to capture, which prompts them to brake, or by aiding in the regulation of swimming speed. Higher swimming velocities have been observed in several species of fish during tracking and striking after the lateral line system has been blocked [44], [88], [89] and detection of self-generated flow fields around the body by the lateral line has been shown to function in other behaviors, such as obstacle avoidance [90]–[93]. Lateral line information also appears to guide the final moments of the strike in blacktip sharks. While intact blacktip sharks rarely missed, blocking the lateral line caused frequent misses associated with high velocity (271±84 cm/s or 3.62±1.10 BL/s) strikes (see Movie S4); successful capture was associated with lower velocity strikes (153±37 cm/s or 1.17±0.26 BL/s, *P* = 0.03) that may not require braking. In the bonnethead, blocking the lateral line did not affect capture success or strike velocity. Since bonnetheads strike with a lower velocity (59±2 cm/s or 0.75±0.03 BL/s), this species may rely less on feedback from self-generated mechanosensory noise to regulate its swimming speed, as slower swimming speeds have been suggested to create less hydrodynamic noise [94], [95]. Swimming velocity as the bonnethead approaches the prey could instead be mediated by their enhanced binocular vision [83]; swimming speed during other behaviors, such as schooling, is visually regulated in other fishes [96]. Additionally, since slower velocity strikes generate smaller bow waves in front of the head, delaying prey escape responses [97], [98], the lateral line may not be critical for strike precision at slower speeds. Nurse sharks, with their very close proximity strikes, may not need lateral line-mediated strike adjustment. Since suction feeders use little to no forward motion, the prey is primarily alerted to the presence of the predator by the suction flow [99], often too late for a successful escape response.

### Capture

**Figure 6:**
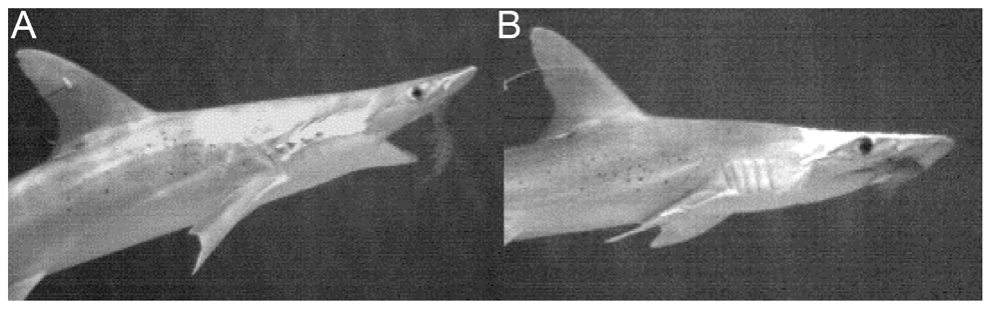
Prey capture in intact vs. electroreception-blocked bonnetheads. A. A bonnethead, *Sphyrna tiburo*, with all senses intact opens the mouth to capture shrimp using ram-biting. B. The same bonnethead fails to open the mouth when electroreception is blocked and misses the shrimp, despite making tactile contact with the prey.

High-speed video analysis has demonstrated that the final milliseconds of a strike leading to capture require precisely timed jaw movements [100] (see Movies S1–S3). In all three species, electroreception triggered jaw depression. Without electroreception, blacktip and nurse sharks missed, unless they touched the prey (with the snout, near the mouth) prior to beginning to move the jaws, which then lead to capture (blacktip shark: Pearson Product Correlation, *p* = 0.00002; nurse shark: Pearson Product Correlation, *p* = 0.00006; see also Movie S5–S8). While repeated strikes in electroreception-blocked blacktip and nurse sharks eventually resulted in capture in all trials, electroreception-blocked bonnetheads failed to open their jaws, despite repeated strikes, and never made successful captures even when touching the prey (Figure 6; see also Movie S9). This suggests that jaw depression is completely guided by the electrical field surrounding the prey and tactile cues were insufficient to initiate jaw movements. The complete reliance on electroreception for prey capture in bonnetheads may be related to the evolution of wider heads in the hammerhead shark family, as their lateral head expansion supports widely spread electroreceptors sampling a large area of the environment [17]. Other enhanced sensory capabilities, such as olfaction, also have been linked to wider heads in sharks [55].

When capture occurs, it is altered in response to sensory deprivation. Ram-feeding blacktip sharks use less ram when vision-blocked but do not change the amount of suction, producing an overall ram feeding event [100]. In contrast, a ram-feeding teleost, the largemouth bass (*Micropterus salmoides*), both decreases ram and actively increases suction when vision-blocked, resulting in a switch from ram to suction feeding [44]. This difference may reflect anatomical limitations on suction generation in the blacktip shark. Unlike many ram-feeding bony fishes, many ram-feeding sharks do not possess the laterally occluded mouth that is characteristic of suction feeders [13]. On the other hand, the nurse shark, a characteristic suction feeder [16], does have the proper mouth morphology to actively increase suction and does so when vision-blocked, while simultaneously decreasing ram [100]. These results suggest that blacktip and nurse sharks modify their capture strategy slightly when using alternate sensory modalities to locate and capture prey. The bonnethead shows little to no change in capture kinematics in response to sensory deprivation, which suggests that capture plasticity varies by species, rather than by capture mechanism [100].

## Summary and Conclusions

This study was designed to link the existing physical knowledge of underwater signal dispersal and the behavioral function of individual senses of sharks during their hunting behavior. It is important to realize that our understanding of signal dispersal is typically based on mathematical models developed from physical experiments under idealized, low-noise, single modality conditions [1], [82], [101]. In nature, conditions can be substantially different and cues can be masked by noise, forcing animals to use the best available information rather than mathematically idealized stimulus fields. Using a large laboratory flume allowed us to test animals under standardized naturalistic conditions and with sensory manipulations that would be nearly impossible to accomplish in the field.

As expected, sensory deprivation confirmed that each sense had its optimal range of use; these ranges overlap to form a smooth sequence from detection to capture (Figure 1A). The olfactory range is large but limited to downstream dispersal where directional information in the odor far-field is difficult to extract [1]. When an attractive odor is detected but the source itself is out of direct detection range, the logical response is to swim upstream [59], [60]. The sharks appear to determine upstream directional information from their drift in the bulk flow. Drift can be measured by the lateral line from boundary layer effects on turbulence [65] and in the case of nurse sharks from actual touch of the (sea) floor [67]. In the flume and in near-bottom habitats, drift can also be measured visually [69] as shown by the effects of sensory blocks. Closer to the source, the prey wake can provide simultaneous odor and turbulence information; this odor near-field becomes increasingly directional [1]. In blacktip sharks and bonnetheads, visual detection of the prey itself can direct the strike from any approach angle, upstream and downstream. The timing then changes from minutes of tracking to (sub-)seconds of striking during which the lateral line and electroreception provide precise information on target location. Finally, capture is most dependent on electroreception to coordinate the 10–100 millisecond-scale ram-suction movements. Touch can occasionally lead to capture when electroreception is blocked. Odor may continue to motivate the behavior: nurse sharks do not hunt at all without odor information, while the more pelagic species still hunt after seeing the prey target, allowing them to strike from upstream directions.

In earlier experiments with food odor sources instead of live prey, we noted on several occasions that sharks after locating the source, but not finding actual food, circled back downstream and repeated the search sequence from a distance, over and over [20]. This gave the impression that the hunting sequence was stereotyped. However, the current results using live prey that emit a variety of sensory cues show that there is considerable plasticity in the behavior and use of senses. We conclude that tracking, the olfactory hunting phase, is indeed stereotyped, although different for each species. This may be due to the lack of precise vector information when only odor and flow are available: the observed stereotyped sequences may have evolved as the optimal solution for the difficult task of odor plume tracking. Then, when direct prey contact has been made, the sharks show plasticity in the use of senses.

Other reports have focused on shark attraction to prey sounds [37], [102]. As stated, we deliberately did not include in this laboratory study the use of sound, particularly directional sound. To avoid reverberation, credible analysis of guidance by sound should be done under field conditions and this will remain an interesting challenge. Our results show the lateral line was clearly involved in directing the strike; this suggests that the sound near-field may have provided directional hydrodynamic information that was not extracted by the otolithic (inertial detection) organs. The lateral line can detect both the wake turbulence [103] and the sound near-field (particle displacement) of the prey [104]; mathematical models suggest that the latter would be expected to provide better information on the precise location of the prey [101].

Sensory hierarchies have been developed for a few other vertebrates, but only for one phase of a behavior, such as prey identification in bats [105], detection in lemurs [106], tracking in catfish [103], striking in snakes [107], and capture in frogs [108]. This study is therefore the first to describe the hierarchy of senses engaged in guiding a complete behavioral sequence in a vertebrate, in this case hunting in aquatic vertebrate predators, focusing on sharks as a model group.

Our results demonstrate that sharks are capable of attending to multiple sensory cues simultaneously, switching sensory modalities in a hierarchical fashion as they approach their prey, and substituting alternate sensory cues, when necessary, to accomplish behavioral tasks. This flexibility in behavior suggests that sharks are well adapted to succeed, even in the face of a changing environment and evolutionary advancements in prey defenses, including chemical, visual, and mechanical camouflage [109], [110]. Such flexibility may have contributed to the success of these marine apex predators and may continue to do so as environments and ecosystems change. Sharks, however, are not unique in their sensory guidance of hunting: they exploit information fields available to all marine species. Thus, the results may be seen as a general blueprint for underwater hunting, modifiable by habitat and by the behavioral specializations of many different aquatic animals from lobsters to whales. These results set the stage for neurobiological analysis of sensory integration in the brains of hunting animals and inspire the design of underwater navigation algorithms for autonomous vehicles requiring plasticity in adapting to unpredictable and variable local conditions [111].

## Supporting Information

Table S1 **Data summary – blacktip shark,** ***Carcharhinus limbatus*** **.** Summary of all variables for the blacktip shark, *Carcharhinus limbatus* with all senses intact, and following blocks of the senses as indicated. Abbreviations: O =  olfaction, V =  vision, L =  lateral line, E =  electroreception. All means are ±s.e.m. The *p* values are the results of linear mixed effects model analyses or Skillings-Mack tests performed on each variable. Value marked (*) are significant after Benjamini-Hochberg corrections. Tukey Test *p* values reflect the results of pairwise post-hoc comparisons between treatments. N.A.: Not applicable, parameter was not assessed because behavior did not occur; N.S.: Non-significant at α = 0.05.
(DOCX)

Table S2 **Data summary – bonnethead,** ***Sphyrna tiburo*** **.** Summary of all variables for the bonnethead, *Sphyrna tiburo* with all senses intact, and following blocks of the senses as indicated. Abbreviations: O =  olfaction, V =  vision, L =  lateral line, E =  electroreception. All means are ±s.e.m. The *p* values are the results of linear mixed effects model analyses or Skillings-Mack tests performed on each variable. Value marked (*) are significant after Benjamini-Hochberg corrections. Tukey Test *p* values reflect the results of pairwise post-hoc comparisons between treatments. N.A.: Not applicable, parameter was not assessed because behavior did not occur; N.R.: Not recorded due to technical difficulties; N.S.: Non-significant at α = 0.05.
(DOCX)

Table S3 **Data summary – nurse shark,** ***Ginglymostoma cirratum*** **.** Summary of all variables for the nurse shark, *Ginglymostoma cirratum* with all senses intact, and following blocks of the senses as indicated. Abbreviations: O =  olfaction, V =  vision, L =  lateral line, E =  electroreception. All means are ±s.e.m. The *p* values are the results of linear mixed effects model analyses or Skillings-Mack tests performed on each variable. Value marked (*) are significant after Benjamini-Hochberg corrections. Tukey Test *p* values reflect the results of pairwise post-hoc comparisons between treatments. N.A.: Not applicable, parameter was not assessed because behavior did not occur; N.S.: Not significant at α = 0.05.
(DOCX)

Movie S1 **Prey capture in an intact blacktip shark,** ***Carcharhinus limbatus*** **, played in slow motion at 30 frames/s (filmed at 250 frames/s).** Rapid (mean: 2.0 BL/s) strikes are initiated from a distance (mean: 240 cm) and the prey is overtaken and engulfed using ram-capture (100% capture success rate).
(AVI)

Movie S2 **Prey capture in an intact bonnethead,** ***Sphyrna tiburo*** **, played in slow motion at 30 frames/s (filmed at 250 frames/s).** Strikes are slower (mean: 0.8 BL/s) and initiated from a closer proximity (mean: 74 cm) than in the blacktip shark. Prey is overtaken and captured between the teeth, using ram-biting (100% capture success rate).
(AVI)

Movie S3 **Prey capture in an intact nurse shark,** ***Ginglymostoma cirratum*** **, played in slow motion at 30 frames/s (filmed at 250 frames/s).** Slow (mean: 0.3 BL/s) strikes, consisting of raising the head, are initiated from close proximity (mean: 13 cm). The prey is rapidly drawn into the mouth using suction (100% capture success rate).
(AVI)

Movie S4 **Prey capture in a blacktip shark,** ***Carcharhinus limbatus*** **, with the lateral line blocked, played in slow motion at 30 frames/s (filmed at 250 frames/s).** When lateral line information is absent, the blacktip sharks frequently missed the prey. These misses were associated with faster strikes (mean: 3.6 BL/s).
(AVI)

Movie S5 **Attempted prey capture (miss) in a blacktip shark,** ***Carcharhinus limbatus*** **, with electroreception blocked, played in slow motion at 30 frames/s (filmed at 250 frames/s).** When electrical cues are absent, blacktip sharks frequently missed unless they touched the prey (with the snout, near the mouth) prior to beginning to move the jaws (see Movie S6 for an example of a successful capture).
(AVI)

Movie S6 **Prey capture (successful) in a blacktip shark,** ***Carcharhinus limbatus*** **, with electroreception blocked, played in slow motion at 30 frames/s (filmed at 250 frames/s).** When electrical cues are absent, blacktip sharks could successfully capture prey if they touched it (with the snout, near the mouth), prior to beginning to move the jaws. If they did not touch the prey, they frequently missed (see Movie S5 for an example of a miss).
(AVI)

Movie S7 **Attempted prey capture (miss) in a nurse shark,** ***Ginglymostoma cirratum*** **, with electroreception blocked, played in slow motion at 30 frames/s (filmed at 250 frames/s).** When electrical cues are absent, nurse sharks frequently missed unless they touched the prey (with the snout, near the mouth) prior to beginning to move the jaws (see Movie S8 for an example of a successful capture).
(AVI)

Movie S8 **Prey capture (successful) in a nurse shark,** ***Ginglymostoma cirratum*** **, with electroreception blocked, played in slow motion at 30 frames/s (filmed at 250 frames/s).** When electrical cues are absent, nurse sharks could successfully capture the prey if they touched it (with the snout, near the mouth) prior to beginning to move the jaws. If they did not touch the prey, they frequently missed (see Movie S7 for an example of a miss).
(AVI)

Movie S9 **Striking behavior in a bonnethead,** ***Sphyrna tiburo*** **, with electroreception blocked, played in slow motion at 30 frames/s (filmed at 250 frames/s).** When electrical cues are absent, the bonnethead fails to open the mouth and misses the prey (0% capture success rate).
(AVI)
