# Supplementary material for: Multisensory Integration and Behavioral Plasticity in Sharks from Different Ecological Niches
Source: PLoS One. 2014 Apr 2;9(4):e93036. doi: 10.1371/journal.pone.0093036 (PMC3973673; doi:10.1371/journal.pone.0093036)
Supplement: Table S1 — Data summary – blacktip shark, Carcharhinus limbatus . Summary of all variables for the blacktip shark, Carcharhinus limbatus with all senses intact, and following blocks of the senses as indicated. Abbreviations: O = olfaction, V = vision, L = lateral line, E = electroreception. All means are ±s.e.m. The p values are the results of linear mixed effects model analyses or Skillings-Mack tests performed on each variable. Value marked (*) are significant after Benjamini-Hochberg corrections. Tukey Test p values reflect the results of pairwise post-hoc comparisons between treatments. N.A.: Not applicable, parameter was not assessed because behavior did not occur; N.S.: Non-significant at α = 0.05. (DOCX) [file pone.0093036.s001.docx]

|  |  |  |  |  | **Tukey Test** | | | | | | | |
| --- | --- | --- | --- | --- | --- | --- | --- | --- | --- | --- | --- | --- |
| **Variable** | **Treatment** | **Mean** | **n** | ***p* value** | **vs. O block** | **vs. V. block** | **vs. O + V block** | **vs. L block** | **vs. L + O block** | **vs. L + V block** | **vs. L + O + V block** | **vs. E. block** |
| Swim Velocity (BL/s) | Control | 1.19±0.03 | 17 | <0.0001* | N.S. | N.S. | <0.001 | N.S. | N.S. | N.S. | <0.001 | N.S. |
|  | O block | 1.10±0.04 | 8 |  |  | N.S. | <0.001 | N.S. | N.S. | N.S. | <0.001 | N.S. |
|  | V block | 0.99±0.06 | 12 |  |  |  | N.S. | N.S. | N.S. | N.S. | N.S. | N.S. |
|  | O + V block | 0.72±0.03 | 9 |  |  |  |  | <0.001 | <0.001 | N.S. | N.S. | <0.001 |
|  | L block | 1.40±0.13 | 5 |  |  |  |  |  | N.S. | <0.001 | <0.001 | N.S. |
|  | L + O block | 1.14±0.05 | 5 |  |  |  |  |  |  | N.S. | <0.001 | N.S. |
|  | L + V block | 0.92±0.06 | 5 |  |  |  |  |  |  |  | N.S. | N.S. |
|  | L + V + O block | 0.70±0.04 | 4 |  |  |  |  |  |  |  |  | <0.001 |
|  | E block | 1.20±0.26 | 3 |  |  |  |  |  |  |  |  |  |
| Turn Velocity (°/s) | Control | 140.2±2.1 | 17 | <0.0001* | <0.001 | N.S. | <0.001 | N.S. | <0.001 | N.S. | <0.001 | N.S. |
|  | O block | 54.3±2.3 | 8 |  |  | <0.001 | N.S. | <0.001 | N.S. | <0.001 | N.S. | <0.001 |
|  | V block | 163.1±6.9 | 12 |  |  |  | <0.001 | N.S. | <0.001 | N.S. | <0.001 | N.S. |
|  | O + V block | 52.9±4.3 | 9 |  |  |  |  | <0.001 | N.S. | <0.001 | N.S. | <0.001 |
|  | L block | 138.8±16.9 | 5 |  |  |  |  |  | <0.001 | N.S. | <0.001 | N.S. |
|  | L + O block | 47.8±7.2 | 5 |  |  |  |  |  |  | <0.001 | N.S. | <0.001 |
|  | L + V block | 110.8±18.9 | 5 |  |  |  |  |  |  |  | 0.002 | N.S. |
|  | L + V + O block | 48.3±3.4 | 4 |  |  |  |  |  |  |  |  | <0.001 |
|  | E block | 163.9±35.1 | 3 |  |  |  |  |  |  |  |  |  |
| Turn Frequency | Control | 0.72±0.04 | 17 | <0.0001* | <0.001 | N.S. | <0.001 | N.S. | <0.001 | <0.001 | <0.001 | N.S. |
| (turns/s) | O block | 0.16±0.03 | 8 |  |  | <0.001 | 0.04 | <0.001 | 0.0087 | <0.001 | N.S. | <0.001 |
|  | V block | 0.88±0.04 | 12 |  |  |  | <0.001 | N.S. | <0.001 | <0.001 | <0.001 | N.S. |
|  | O + V block | 0.20±0.01 | 9 |  |  |  |  | <0.001 | N.S. | 0.002 | N.S. | <0.001 |
|  | L block | 0.56±0.08 | 5 |  |  |  |  |  | <0.001 | N.S. | <0.001 | N.S. |
|  | L + O block | 0.23±0.03 | 5 |  |  |  |  |  |  | N.S. | N.S. | <0.001 |
|  | L + V block | 0.36±0.03 | 5 |  |  |  |  |  |  |  | <0.001 | <0.001 |
|  | L + V + O block | 0.16±0.01 | 4 |  |  |  |  |  |  |  |  | <0.001 |
|  | E block | 1.08±0.01 | 3 |  |  |  |  |  |  |  |  |  |
| Tracking Time (s) | Control | 35.0±9.5 | 17 | 0.0003* |  | <0.001 |  | 0.03 |  |  |  | N.S. |
|  | O block | N.A. | 8 |  |  |  |  |  |  |  |  |  |
|  | V block | 949.3±236.1 | 12 |  |  |  |  | <0.001 |  |  |  | 0.02 |
|  | O + V block | N.A. | 9 |  |  |  |  |  |  |  |  |  |
|  | L block | 13.1±5.4 | 5 |  |  |  |  |  |  |  |  | N.S. |
|  | L + O block | N.A. | 5 |  |  |  |  |  |  |  |  |  |
|  | L + V block | N.A. | 5 |  |  |  |  |  |  |  |  |  |
|  | L + V + O block | N.A. | 4 |  |  |  |  |  |  |  |  |  |
|  | E block | 106.4±6.2 | 3 |  |  |  |  |  |  |  |  |  |
| Orientation Distance | Control | 238.9±16.3 | 17 | <0.0001* | N.S. | <0.001 |  | N.S. | N.S. |  |  | N.S. |
| (cm) | O block | 253.7±2.5 | 8 |  |  | <0.001 |  | N.S. | N.S. |  |  | N.S. |
|  | V block | 17.4±2.5 | 12 |  |  |  |  | <0.001 | <0.001 |  |  | <0.001 |
|  | O + V block | N.A. | 9 |  |  |  |  |  |  |  |  |  |
|  | L block | 304.8±32.1 | 5 |  |  |  |  |  | 0.02 |  |  | N.S. |
|  | L + O block | 177.5±27.5 | 5 |  |  |  |  |  |  |  |  | N.S. |
|  | L + V block | N.A. | 5 |  |  |  |  |  |  |  |  |  |
|  | L + V + O block | N.A. | 4 |  |  |  |  |  |  |  |  |  |
|  | E block | 214.3±16.3 | 3 |  |  |  |  |  |  |  |  |  |
| Strike Rate | Control | 100.0±0.0 | 17 | <0.0001* | N.S. | <0.05 | <0.001 | N.S. | N.S. | <0.001 | <0.001 | N.S. |
| (%) | O block | 100.0±0.0 | 8 |  |  | <0.05 | <0.001 | N.S. | N.S. | <0.01 | <0.001 | N.S. |
|  | V block | 60.4±11.1 | 12 |  |  |  | <0.05 | <0.05 | <0.05 | <0.05 | <0.05 | <0.05 |
|  | O + V block | 0.0±0.0 | 9 |  |  |  |  | <0.001 | <0.001 | N.S. | N.S. | <0.001 |
|  | L block | 100.0±0.0 | 5 |  |  |  |  |  | N.S. | <0.001 | <0.001 | N.S. |
|  | L + O block | 100.0±0.0 | 5 |  |  |  |  |  |  | <0.001 | <0.001 | N.S. |
|  | L + V block | 0.0±0.0 | 5 |  |  |  |  |  |  |  | N.S. | <0.001 |
|  | L + V + O block | 0.0±0.0 | 4 |  |  |  |  |  |  |  |  | <0.001 |
|  | E block | 100.0±0.0 | 3 |  |  |  |  |  |  |  |  |  |
| Strike Angle (°) | Control | 15.4±2.5 | 17 | <0.0001* | N.S. | <0.001 |  | N.S. | N.S. |  |  | N.S. |
|  | O block | 17.6±3.9 | 8 |  |  | <0.001 |  | N.S. | N.S. |  |  | N.S. |
|  | V block | 91.7±15.5 | 12 |  |  |  |  | <0.001 | 0.02 |  |  | N.S. |
|  | O + V block | N.A. | 9 |  |  |  |  |  |  |  |  |  |
|  | L block | 7.7±11 | 5 |  |  |  |  |  | N.S. |  |  | N.S. |
|  | L + O block | 21.7±5.6 | 5 |  |  |  |  |  |  |  |  | N.S. |
|  | L + V block | N.A. | 5 |  |  |  |  |  |  |  |  |  |
|  | L + V + O block | N.A. | 4 |  |  |  |  |  |  |  |  |  |
|  | E block | 23.5±7.8 | 3 |  |  |  |  |  |  |  |  |  |
| Strike Velocity (BL/s) | Control | 1.95±0.21 | 17 | 0.01* | N.S. | 0.001 |  | N.S. | N.S. | N.S. |  | N.S. |
|  | O block | 1.50±0.04 | 8 |  |  | N.S. |  | N.S. | N.S. | N.S. |  | N.S. |
|  | V block | 1.25±0.31 | 12 |  |  |  |  | 0.002 | N.S. | N.S. |  | N.S. |
|  | O + V block | N.A. | 9 |  |  |  |  |  |  |  |  |  |
|  | L block | 2.88±0.86 | 5 |  |  |  |  |  | N.S. | N.S. |  | N.S. |
|  | L + O block | 1.40±0.08 | 5 |  |  |  |  |  |  |  |  | N.S. |
|  | L + V block | N.A. | 5 |  |  |  |  |  |  |  |  |  |
|  | L + V + O block | N.A. | 4 |  |  |  |  |  |  |  |  |  |
|  | E block | 1.59±0.06 | 3 |  |  |  |  |  |  |  |  |  |
| Number of Misses | Control | 0.00±0.00 | 17 | <0.0001* | N.S. | N.S. |  | <0.05 | <0.01 |  |  | <0.001 |
|  | O block | 0.00±0.00 | 8 |  |  | N.S. |  | <0.05 | <0.05 |  |  | <0.05 |
|  | V block | 0.11±0.05 | 12 |  |  |  |  | N.S. | N.S. |  |  | N.S. |
|  | O + V block | N.A. | 9 |  |  |  |  |  |  |  |  |  |
|  | L block | 0.34±0.19 | 5 |  |  |  |  |  | N.S. |  |  | N.S. |
|  | L + O block | 0.58±0.23 | 5 |  |  |  |  |  |  |  |  | N.S. |
|  | L + V block | N.A. | 5 |  |  |  |  |  |  |  |  |  |
|  | L + V + O block | N.A. | 4 |  |  |  |  |  |  |  |  |  |
|  | E block | 1.17±0.58 | 3 |  |  |  |  |  |  |  |  |  |
| Capture Success | Control | 100.0±0.0 | 17 | <0.0001* | N.S. | <0.05 |  | N.S. | N.S. |  |  | N.S. |
| Rate (%) | O block | 100.0±0.0 | 8 |  |  | <0.05 |  | N.S. | N.S. |  |  | N.S. |
|  | V block | 50.0±10.5 | 12 |  |  |  |  | <0.05 | <0.05 |  |  | <0.05 |
|  | O + V block | N.A. | 9 |  |  |  |  |  |  |  |  |  |
|  | L block | 100.0±0.0 | 5 |  |  |  |  |  | N.S. |  |  | N.S. |
|  | L + O block | 100.0±0.0 | 5 |  |  |  |  |  |  |  |  | N.S. |
|  | L + V block | N.A. | 5 |  |  |  |  |  |  |  |  |  |
|  | L + V + O block | N.A. | 4 |  |  |  |  |  |  |  |  |  |
|  | E block | 100.0±0.0 | 3 |  |  |  |  |  |  |  |  |  |
