# Supplementary material for: Multisensory Integration and Behavioral Plasticity in Sharks from Different Ecological Niches
Source: PLoS One. 2014 Apr 2;9(4):e93036. doi: 10.1371/journal.pone.0093036 (PMC3973673; doi:10.1371/journal.pone.0093036)
Supplement: Table S2 — Data summary – bonnethead, Sphyrna tiburo . Summary of all variables for the bonnethead, Sphyrna tiburo with all senses intact, and following blocks of the senses as indicated. Abbreviations: O = olfaction, V = vision, L = lateral line, E = electroreception. All means are ±s.e.m. The p values are the results of linear mixed effects model analyses or Skillings-Mack tests performed on each variable. Value marked (*) are significant after Benjamini-Hochberg corrections. Tukey Test p values reflect the results of pairwise post-hoc comparisons between treatments. N.A.: Not applicable, parameter was not assessed because behavior did not occur; N.R.: Not recorded due to technical difficulties; N.S.: Non-significant at α = 0.05. (DOCX) [file pone.0093036.s002.docx]

|  |  |  |  |  | **Tukey Test** | | | | | | |
| --- | --- | --- | --- | --- | --- | --- | --- | --- | --- | --- | --- |
| **Variable** | **Treatment** | **Mean** | **n** | ***p* value** | **vs. O block** | **vs. V. block** | **vs. O + V block** | **vs. L block** | **vs. L + O block** | **vs. L + V block** | **vs. E. block** |
| Swim Velocity (BL/s) | Control | 0.69±0.02 | 14 | 0.009* | N.S. | N.S. | N.S. | N.S. |  | 0.009 | N.S. |
|  | O block | 0.79±0.04 | 5 |  |  | N.S. | N.S. | N.S. |  | <0.001 | N.S. |
|  | V block | 0.71±0.00 | 4 |  |  |  | N.S. | N.S. |  | N.S. | N.S. |
|  | O + V block | 0.74±0.00 | 2 |  |  |  |  | N.S. |  | N.S. | N.S. |
|  | L block | 0.74±0.05 | 4 |  |  |  |  |  |  | <0.001 | N.S. |
|  | L + O block | N.R. | 3 |  |  |  |  |  |  |  |  |
|  | L + V block | 0.49±0.03 | 3 |  |  |  |  |  |  |  | 0.002 |
|  | E block | 0.75±0.06 | 6 |  |  |  |  |  |  |  |  |
| Turn Velocity (°/s) | Control | 137.4±5.7 | 14 | <0.0001* | <0.001 | N.S. | <0.001 | 0.01 |  | 0.03 | N.S. |
|  | O block | 47.2±4.9 | 5 |  |  | <0.001 | N.S. | <0.001 |  | <0.001 | <0.001 |
|  | V block | 128.0±0.0 | 4 |  |  |  | <0.001 | N.S. |  | N.S. | N.S. |
|  | O + V block | 38.7±0.0 | 2 |  |  |  |  | <0.001 |  | <0.001 | <0.001 |
|  | L block | 98.7±3.6 | 4 |  |  |  |  |  |  | N.S. | 0.01 |
|  | L + O block | N.R. | 3 |  |  |  |  |  |  |  |  |
|  | L + V block | 100.3±13.0 | 3 |  |  |  |  |  |  |  | 0.03 |
|  | E block | 148.7±4.6 | 6 |  |  |  |  |  |  |  |  |
| Turn Frequency | Control | 0.81±0.04 | 14 | <0.0001* | <0.001 | N.S. | <0.001 | N.S. |  | <0.001 | N.S. |
| (turns/s) | O block | 0.13±0.01 | 5 |  |  | <0.001 | N.S. | <0.001 |  | <0.001 | <0.001 |
|  | V block | 0.85±0.00 | 4 |  |  |  | <0.001 | N.S. |  | 0.003 | N.S. |
|  | O + V block | 0.10±0.00 | 2 |  |  |  |  | <0.001 |  | <0.001 | <0.001 |
|  | L block | 0.69±0.08 | 4 |  |  |  |  |  |  | N.S. | N.S. |
|  | L + O block | N.R. | 3 |  |  |  |  |  |  |  |  |
|  | L + V block | 0.47±0.07 | 3 |  |  |  |  |  |  |  | <0.001 |
|  | E block | 0.77±0.03 | 6 |  |  |  |  |  |  |  |  |
| Tracking Time (s) | Control | 74.3±18.0 | 14 | 0.04 |  |  |  |  |  |  |  |
|  | O block | N.A. | 5 |  |  |  |  |  |  |  |  |
|  | V block | N.A. | 4 |  |  |  |  |  |  |  |  |
|  | O + V block | N.A. | 2 |  |  |  |  |  |  |  |  |
|  | L block | 17.74±6.1 | 4 |  |  |  |  |  |  |  |  |
|  | L + O block | N.A. | 3 |  |  |  |  |  |  |  |  |
|  | L + V block | N.A. | 3 |  |  |  |  |  |  |  |  |
|  | E block | 41.8±23.5 | 6 |  |  |  |  |  |  |  |  |
| Orientation Distance | Control | 73.8±10.7 | 14 | 0.0008* | <0.001 |  |  | N.S. |  |  | N.S. |
| (cm) | O block | 165.7±31.8 | 5 |  |  |  |  | 0.004 |  |  | 0.002 |
|  | V block | N.A. | 4 |  |  |  |  |  |  |  |  |
|  | O + V block | N.A. | 2 |  |  |  |  |  |  |  |  |
|  | L block | 116.6±16.0 | 4 |  |  |  |  |  |  |  | N.S. |
|  | L + O block | N.R. | 3 |  |  |  |  |  |  |  |  |
|  | L + V block | N.A. | 3 |  |  |  |  |  |  |  |  |
|  | E block | 106.7±22.8 | 6 |  |  |  |  |  |  |  |  |
| Strike Rate (%) | Control | 100.0±0.0 | 14 | <0.0001* | N.S. | <0.001 | <0.001 | N.S. | N.S. | <0.001 | N.S. |
|  | O block | 100.0±0.0 | 5 |  |  | <0.001 | <0.001 | N.S. | N.S. | <0.001 | N.S. |
|  | V block | 0.0±0.0 | 4 |  |  |  | N.S. | <0.001 | <0.001 | N.S. | <0.001 |
|  | O + V block | 0.0±0.0 | 2 |  |  |  |  | <0.001 | <0.001 | N.S. | <0.001 |
|  | L block | 100.0±0.0 | 4 |  |  |  |  |  | N.S. | <0.001 | N.S. |
|  | L + O block | 100.0±0.0 | 3 |  |  |  |  |  |  | <0.001 | N.S. |
|  | L + V block | 0.0±0.0 | 3 |  |  |  |  |  |  |  | <0.001 |
|  | E block | 100.0±0.0 | 6 |  |  |  |  |  |  |  |  |
| Strike Velocity (BL/s) | Control | 0.75±0.03 | 14 | 0.026 |  |  |  |  |  |  |  |
|  | O block | 0.84±0.04 | 5 |  |  |  |  |  |  |  |  |
|  | V block | N.A. | 4 |  |  |  |  |  |  |  |  |
|  | O + V block | N.A. | 2 |  |  |  |  |  |  |  |  |
|  | L block | 0.79±0.05 | 4 |  |  |  |  |  |  |  |  |
|  | L + O block | N.R. | 3 |  |  |  |  |  |  |  |  |
|  | L + V block | N.A. | 3 |  |  |  |  |  |  |  |  |
|  | E block | 0.95±0.08 | 6 |  |  |  |  |  |  |  |  |
| Strike Angle (°) | Control | 28.1±5.1 | 14 | 0.866 |  |  |  |  |  |  |  |
|  | O block | 26.5±8.9 | 5 |  |  |  |  |  |  |  |  |
|  | V block | N.A. | 4 |  |  |  |  |  |  |  |  |
|  | O + V block | N.A. | 2 |  |  |  |  |  |  |  |  |
|  | L block | 30.2±5.5 | 4 |  |  |  |  |  |  |  |  |
|  | L + O block | N.R. | 3 |  |  |  |  |  |  |  |  |
|  | L + V block | N.A. | 3 |  |  |  |  |  |  |  |  |
|  | E block | 25.6±5.6 | 6 |  |  |  |  |  |  |  |  |
| Number of Misses | Control | 0.04±0.04 | 14 | 0.006* | N.S. |  |  | N.S. | N.S. |  | <0.001 |
|  | O block | 0.00±0.00 | 5 |  |  |  |  | N.S. | N.S. |  | <0.001 |
|  | V block | N.A. | 4 |  |  |  |  |  |  |  |  |
|  | O + V block | N.A. | 2 |  |  |  |  |  |  |  |  |
|  | L block | 0.25±0.14 | 4 |  |  |  |  |  | N.S. |  | <0.01 |
|  | L + O block | 0.25±0.25 | 3 |  |  |  |  |  |  |  | <0.01 |
|  | L + V block | N.A. | 3 |  |  |  |  |  |  |  |  |
|  | E block | 35.4±11.7 | 6 |  |  |  |  |  |  |  |  |
| Capture Success | Control | 100.0±0.0 | 14 | <0.0001* | N.S. |  |  | N.S. | N.S. |  | <0.001 |
| Rate (%) | O block | 100.0±0.0 | 5 |  |  |  |  | N.S. | N.S. |  | <0.001 |
|  | V block | N.A. | 4 |  |  |  |  |  |  |  |  |
|  | O + V block | N.A. | 2 |  |  |  |  |  |  |  |  |
|  | L block | 100.0±0.0 | 4 |  |  |  |  |  | N.S. |  | <0.001 |
|  | L + O block | 100.0±0.0 | 3 |  |  |  |  |  |  |  | <0.001 |
|  | L + V block | 100.0±0.0 | 3 |  |  |  |  |  |  |  |  |
|  | E block | 0.0±0.0 | 6 |  |  |  |  |  |  |  |  |
