# Supplementary material for: Multisensory Integration and Behavioral Plasticity in Sharks from Different Ecological Niches
Source: PLoS One. 2014 Apr 2;9(4):e93036. doi: 10.1371/journal.pone.0093036 (PMC3973673; doi:10.1371/journal.pone.0093036)
Supplement: Table S3 — Data summary – nurse shark, Ginglymostoma cirratum . Summary of all variables for the nurse shark, Ginglymostoma cirratum with all senses intact, and following blocks of the senses as indicated. Abbreviations: O = olfaction, V = vision, L = lateral line, E = electroreception. All means are ±s.e.m. The p values are the results of linear mixed effects model analyses or Skillings-Mack tests performed on each variable. Value marked (*) are significant after Benjamini-Hochberg corrections. Tukey Test p values reflect the results of pairwise post-hoc comparisons between treatments. N.A.: Not applicable, parameter was not assessed because behavior did not occur; N.S.: Not significant at α = 0.05. (DOCX) [file pone.0093036.s003.docx]

|  |  |  |  |  | **Tukey Test** | | | | | | | |
| --- | --- | --- | --- | --- | --- | --- | --- | --- | --- | --- | --- | --- |
| **Variable** | **Treatment** | **Mean** | **n** | ***p* value** | **vs. O block** | **vs. V. block** | **vs. O + V block** | **vs. L block** | **vs. L + O block** | **vs. L + V block** | **vs. L + O + V block** | **vs. E. block** |
| Swim Velocity (BL/s) | Control | 0.44±0.04 | 7 | <0.0001* | N.S. | N.S. | N.S. | <0.001 | 0.009 | N.S. | N.S. | N.S. |
|  | O block | 0.54±0.06 | 7 |  |  | N.S. | N.S. | N.S. | N.S. | N.S. | N.S. | N.S. |
|  | V block | 0.35±0.01 | 7 |  |  |  | N.S. | <0.001 | <0.001 | N.S. | N.S. | 0.006 |
|  | O + V block | 0.41±0.05 | 5 |  |  |  |  | <0.001 | 0.002 | N.S. | N.S. | N.S. |
|  | L block | 0.76±0.07 | 6 |  |  |  |  |  | N.S. | 0.01 | N.S. | N.S. |
|  | L + O block | 0.73±0.08 | 5 |  |  |  |  |  |  | N.S. | N.S. | N.S. |
|  | L + V block | 0.46±0.02 | 6 |  |  |  |  |  |  |  | N.S. | N.S. |
|  | L + V + O block | 0.53±0.02 | 5 |  |  |  |  |  |  |  |  | N.S. |
|  | E block | 0.60±0.02 | 6 |  |  |  |  |  |  |  |  |  |
| Turn Velocity (°/s) | Control | 83.6±4.8 | 7 | <0.0001* | <0.001 | N.S. | <0.001 | N.S. | <0.001 | N.S. | <0.001 | N.S. |
|  | O block | 47.8±2.9 | 7 |  |  | <0.001 | N.S. | N.S. | N.S. | <0.001 | N.S. | 0.002 |
|  | V block | 83.8±4.2 | 7 |  |  |  | <0.001 | N.S. | <0.001 | N.S. | <0.001 | N.S. |
|  | O + V block | 40.0±2.6 | 5 |  |  |  |  | 0.002 | N.S. | <0.001 | N.S. | <0.001 |
|  | L block | 62.1±3.3 | 6 |  |  |  |  |  | N.S. | N.S. | N.S. | N.S. |
|  | L + O block | 45.2±7.9 | 5 |  |  |  |  |  |  | <0.001 | N.S. | <0.001 |
|  | L + V block | 74.8±3.6 | 6 |  |  |  |  |  |  |  | 0.002 | N.S. |
|  | L + V + O block | 47.5±3.1 | 5 |  |  |  |  |  |  |  |  | 0.005 |
|  | E block | 73.9±5.9 | 6 |  |  |  |  |  |  |  |  |  |
| Turn Frequency | Control | 0.52±0.03 | 7 | <0.0001* | <0.001 | N.S. | <0.001 | 0.03 | <0.001 | N.S. | <0.001 | N.S. |
| (turns/s) | O block | 0.11±0.01 | 7 |  |  | <0.001 | N.S. | <0.001 | N.S. | <0.001 | N.S. | <0.001 |
|  | V block | 0.49±0.03 | 7 |  |  |  | <0.001 | N.S. | <0.001 | N.S. | <0.001 | N.S. |
|  | O + V block | 0.15±0.02 | 5 |  |  |  |  | <0.01 | N.S. | <0.001 | N.S. | <0.001 |
|  | L block | 0.31±0.04 | 6 |  |  |  |  |  | <0.001 | N.S. | N.S. | N.S. |
|  | L + O block | 0.11±0.02 | 5 |  |  |  |  |  |  | <0.001 | <0.001 | <0.001 |
|  | L + V block | 0.41±0.04 | 6 |  |  |  |  |  |  |  | <0.001 | N.S. |
|  | L + V + O block | 0.17±0.03 | 5 |  |  |  |  |  |  |  |  | <0.001 |
|  | E block | 0.45±0.07 | 6 |  |  |  |  |  |  |  |  |  |
| Tracking Time (s) | Control | 98.4±20.6 | 7 | <0.0001* |  | N.S. |  | <0.001 |  | N.S. |  | N.S. |
|  | O block | N.A. | 7 |  |  |  |  |  |  |  |  |  |
|  | V block | 99.4±15.7 | 7 |  |  |  |  | 0.04 |  | N.S. |  | N.S. |
|  | O + V block | N.A. | 5 |  |  |  |  |  |  |  |  |  |
|  | L block | 17.6±4.5 | 6 |  |  |  |  |  |  | <0.001 |  | 0.04 |
|  | L + O block | N.A. | 5 |  |  |  |  |  |  |  |  |  |
|  | L + V block | 189.8±72.2 | 6 |  |  |  |  |  |  |  |  | 0.004 |
|  | L + V + O block | N.A. | 5 |  |  |  |  |  |  |  |  |  |
|  | E block | 52.43±15.5 | 6 |  |  |  |  |  |  |  |  |  |
| Resting Time (%) | Control | 0.2±0.2 | 7 | <0.0001* | <0.001 | N.S. | <0.001 | N.S. | N.S. | N.S. | 0.01 | N.S. |
|  | O block | 50.9±12.2 | 7 |  |  | <0.001 | N.S. | <0.001 | N.S. | <0.001 | N.S. | <0.001 |
|  | V block | 0.0±0.0 | 7 |  |  |  | <0.001 | N.S. | N.S. | N.S. | 0.01 | N.S. |
|  | O + V block | 75.4±37.2 | 5 |  |  |  |  | <0.001 | 0.03 | <0.001 | N.S. | <0.001 |
|  | L block | 0.0±0.0 | 6 |  |  |  |  |  | N.S. | N.S. | 0.02 | N.S. |
|  | L + O block | 29.5±.12.0 | 5 |  |  |  |  |  |  | N.S. | N.S. | N.S. |
|  | L + V block | 0.0±0.0 | 6 |  |  |  |  |  |  |  | 0.02 | N.S. |
|  | L + V + O block | 41.1±15.5 | 5 |  |  |  |  |  |  |  |  | N.S. |
|  | E block | 3.2±3.2 | 6 |  |  |  |  |  |  |  |  |  |
| Orientation Distance | Control | 12.7±1.4 | 7 | 0.328 |  |  |  |  |  |  |  |  |
| (cm) | O block | 13.9±1.1 | 7 |  |  |  |  |  |  |  |  |  |
|  | V block | 15.2±2.2 | 7 |  |  |  |  |  |  |  |  |  |
|  | O + V block | 17.7±0.0 | 5 |  |  |  |  |  |  |  |  |  |
|  | L block | 20.4±3.3 | 6 |  |  |  |  |  |  |  |  |  |
|  | L + O block | 11.3±2.2 | 5 |  |  |  |  |  |  |  |  |  |
|  | L + V block | 12.8±1.5 | 6 |  |  |  |  |  |  |  |  |  |
|  | L + V + O block | 20.3±0.0 | 5 |  |  |  |  |  |  |  |  |  |
|  | E block | 28.6±6.5 | 6 |  |  |  |  |  |  |  |  |  |
| Strike Rate (%) | Control | 100.0±0.0 | 7 | <0.0001* | <0.001 | N.S. | <0.001 | N.S. | <0.001 | N.S. | <0.001 | N.S. |
|  | O block | 0.0±0.0 | 7 |  |  | <0.001 | N.S. | <0.001 | N.S. | <0.001 | N.S. | <0.001 |
|  | V block | 100.0±0.0 | 7 |  |  |  | <0.001 | N.S. | <0.001 | N.S. | <0.001 | N.S. |
|  | O + V block | 0.0±0.0 | 5 |  |  |  |  | <0.001 | N.S. | <0.001 | N.S. | <0.001 |
|  | L block | 100.0±0.0 | 6 |  |  |  |  |  | <0.001 | N.S. | <0.001 | N.S. |
|  | L + O block | 36.7±0.10 | 5 |  |  |  |  |  |  | <0.001 | N.S. | <0.001 |
|  | L + V block | 100.0±0.0 | 6 |  |  |  |  |  |  |  | <0.001 | N.S. |
|  | L + V + O block | 0.0±0.0 | 5 |  |  |  |  |  |  |  |  | <0.001 |
|  | E block | 100.0±0.0 | 6 |  |  |  |  |  |  |  |  |  |
| Strike Angle (°) | Control | 58.4±10.4 | 7 | 0.006* |  | N.S. |  | N.S. | N.S. | N.S. |  | N.S. |
|  | O block | N.A. | 7 |  |  |  |  |  |  |  |  |  |
|  | V block | 67.4±14.5 | 7 |  |  |  |  | N.S. | N.S. | N.S. |  | N.S. |
|  | O + V block | N.A. | 5 |  |  |  |  |  |  |  |  |  |
|  | L block | 35.8±5.1 | 6 |  |  |  |  |  | N.S. | 0.01 |  | N.S. |
|  | L + O block | 91.9±22.8 | 5 |  |  |  |  |  |  | N.S. |  | 0.03 |
|  | L + V block | 93.7±6.3 | 6 |  |  |  |  |  |  |  |  | 0.004 |
|  | L + V + O block | N.A. | 5 |  |  |  |  |  |  |  |  |  |
|  | E block | 38.4±10.5 | 6 |  |  |  |  |  |  |  |  |  |
| Strike Velocity (BL/s) | Control | 0.31±0.04 | 7 | 0.001* |  | N.S. |  | N.S. |  | N.S. |  | N.S. |
|  | O block | N.A. | 7 |  |  |  |  |  |  |  |  |  |
|  | V block | 0.23±0.06 | 7 |  |  |  |  | 0.002 |  | N.S. |  | <0.001 |
|  | O + V block | N.A. | 5 |  |  |  |  |  |  |  |  |  |
|  | L block | 0.44±0.06 | 6 |  |  |  |  |  |  | 0.004 |  | N.S. |
|  | L + O block | N.A. | 5 |  |  |  |  |  |  |  |  |  |
|  | L + V block | 0.23±0.03 | 6 |  |  |  |  |  |  |  |  | 0.002 |
|  | L + V + O block | N.A. | 5 |  |  |  |  |  |  |  |  |  |
|  | E block | 0.44±0.04 | 6 |  |  |  |  |  |  |  |  |  |
| Number of Misses | Control | 0.00±0.00 | 7 | 0.151 |  |  |  |  |  |  |  |  |
|  | O block | N.A. | 7 |  |  |  |  |  |  |  |  |  |
|  | V block | 0.05±0.05 | 7 |  |  |  |  |  |  |  |  |  |
|  | O + V block | N.A. | 5 |  |  |  |  |  |  |  |  |  |
|  | L block | 0.04±0.04 | 6 |  |  |  |  |  |  |  |  |  |
|  | L + O block | 0.13±0.13 | 5 |  |  |  |  |  |  |  |  |  |
|  | L + V block | 0.00±0.00 | 6 |  |  |  |  |  |  |  |  |  |
|  | L + V + O block | N.A. | 5 |  |  |  |  |  |  |  |  |  |
|  | E block | 0.47±0.26 | 6 |  |  |  |  |  |  |  |  |  |
| Capture Success | Control | 100.0±0.0 | 7 | <0.0001* |  | N.S. |  | N.S. | <0.001 | N.S. |  | N.S. |
| Rate (%) | O block | N.A. | 7 |  |  |  |  |  |  |  |  |  |
|  | V block | 100.0±0.0 | 7 |  |  |  |  | N.S. | <0.001 | N.S. |  | N.S. |
|  | O + V block | N.A. | 5 |  |  |  |  |  |  |  |  |  |
|  | L block | 100.0±0.00 | 6 |  |  |  |  |  | <0.001 | N.S. |  | N.S. |
|  | L + O block | 45.8±4.17 | 5 |  |  |  |  |  |  | <0.001 |  | <0.001 |
|  | L + V block | 100.0±0.0 | 6 |  |  |  |  |  |  |  |  | N.S. |
|  | L + V + O block | N.A. | 5 |  |  |  |  |  |  |  |  |  |
|  | E block | 94.44±5.56 | 6 |  |  |  |  |  |  |  |  |  |
